# Evaluation of Near Infrared Dyes as Markers of P-Glycoprotein Activity in Tumors

**DOI:** 10.3389/fphar.2016.00426

**Published:** 2016-11-15

**Authors:** Inessa Semenenko, Emma Portnoy, Mohammed Aboukaoud, Serge Guzy, Miriam Shmuel, Gal Itzhak, Sara Eyal

**Affiliations:** ^1^Institute for Drug Research, School of Pharmacy, The Hebrew University of JerusalemJerusalem, Israel; ^2^Department of Pharmacometrics, University of Maryland, College ParkMD, USA; ^3^Department of Pharmacometrics, University of Minnesota, MinneapolisMN, USA

**Keywords:** cancer, multidrug resistance, MDR1, P-glycoprotein, indocyanine green, IR-783, near infrared imaging

## Abstract

**Aim:** The multidrug resistance protein 1 (MDR1; P-glycoprotein) has been associated with eﬄux of chemotherapeutic agents from tumor cells and with poor patient prognosis. This study evaluated the feasibility of non-invasive, non-radioactive near infrared (NIR) imaging methodology for detection of MDR1 functional activity in tumors.

**Methods:** Initial accumulation assays were conducted in MDR1-overexpressing MDCK cells (MDCK-MDR1) and control MDCK cells (MDCK-CT) using the NIR dyes indocyanine green (ICG), IR-783, IR-775, rhodamine 800, XenoLight DiR, and Genhance 750, at 0.4 μM–100 μM. ICG and IR-783 were also evaluated in HT-29 cells in which MDR1 overexpression was induced by colchicine (HT-29-MDR1) and their controls (HT-29-CT). *In vivo* optical imaging studies were conducted using immunodeficient mice bearing HT-29-CT and HT-29-MDR1 xenografts.

**Results:** ICG’s emission intensity was 2.0- and 2.2-fold higher in control versus MDR1-overexpressing cells, in MDCK and HT-29 cell lines, respectively. The respective IR-783 control:MDR1 ratio was 1.4 in both MDCK and HT-29 cells. Optical imaging of mice bearing HT-29-CT and HT-29-MDR1 xenografts revealed a statistically non-significant, 1.7-fold difference (*p* > 0.05) in ICG emission intensity between control and MDR1 tumors. No such differences were observed with IR-783.

**Conclusion:** ICG and IR-783 appear to be weak MDR1 substrates. *In vivo*, low sensitivity and high between-subject variability impair the ability to use the currently studied probes as markers of tumor MDR1 activity. The results suggest that, for future use of this technology, additional NIR probes should be screened as MDR1 substrates.

## Introduction

Multidrug resistance (MDR) to chemotherapy remains a clinically challenging issue. Among the mechanisms of MDR, the most common relies on drug eﬄux from tumor cells, mediated by ATP-binding cassette (ABC) transporters (Szakács et al., 2006; Holohan et al., 2013; Zhang et al., 2015). The best studied ABC transporter is the multidrug resistance protein MDR1 [P-glycoprotein (P-gp)], encoded in humans by *ABCB1* (Gottesman and Pastan, 1993; Schinkel et al., 1997). MDR1 substrates include drugs central to most chemotherapeutic regimens, such as vinca alkaloids, anthracyclines, taxanes, epipodophyllotoxins, and tyrosine kinase inhibitors as well as compounds of other pharmacological classes (Mann et al., 2015). In addition, MDR1 expression in a tumor may be a poor prognostic indicator, representative of a more aggressive phenotype (Amiri-Kordestani et al., 2012).

The association between MDR1 expression and patient survival or response to chemotherapy has been best demonstrated in patients with hematological malignancies (Szakács et al., 2006). In adult acute myelogenous leukemia (AML), MDR1 expression was found to be an independent prognostic variable related to induction failure (Pallis and Russell, 2004; van der Holt et al., 2005). Evidence for an association between MDR1 expression and poor clinical outcome exists also for solid tumors, such as breast cancer and sarcoma (Szakács et al., 2006). However, in contrast to hematological malignancies, solid tumors are much more difficult to collect and study. Only in recent years, studies of MDR1 activity in solid tumors have become more feasible, with the introduction of non-invasive imaging techniques and the use of radiolabeled substrates such as ^99m^Tc-methoxyisobutylisonitrile (^99m^Tc-MIBI) and ^11^C-verapamil (Piwnica-Worms et al., 1993; Chen et al., 1997; Gottesman et al., 2002; Del Vecchio et al., 2003; Eary et al., 2011). Nevertheless, PET and SPECT scans are associated with health risk since they involve ionizing radiation, are technically demanding and are costly (James and Gambhir, 2012; Mann et al., 2015, 2016).

An emerging alternative technique for *in vivo* tumor imaging is NIR imaging (Arranz and Ripoll, 2015; Mann et al., 2016). Compared to other optical imaging methods, NIR is characterized by relatively deep tissue penetration, low toxicity, and high signal to noise ratio (Mann et al., 2015, 2016). NIR has been applied to the imaging of breast cancer lesions and sentinel lymph node mapping in prostate and breast cancers, as well as other tumor types (Sevick-Muraca, 2012). The utilization of NIR for imaging MDR1 activity was first suggested by On et al. (2011), with rhodamine 800 as the substrate. Later, we found that the only FDA-approved NIR molecule, ICG, is an MDR1 substrate (Portnoy et al., 2012). Hence, the goal of the current study was to evaluate the feasibility of NIR imaging for identification of MDR1 overexpression in solid tumors.

## Materials and Methods

### Materials

Indocyanine green was purchased from Acros Organics (Geel, Belgium). Cell culture reagents were purchased from Biological Industries (Beit Haemek, Israel). The RNeasy mini-isolation kit was from Qiagen (Valencia, CA, USA). TaqMan reverse transcription reagents and fluorescent MGB probes were from Applied Biosystems (Foster City, CA, USA). Primary antibodies for β-actin and C219 were from Biotest (Kfar Saba, Israel) and the primary antibody for OATP 1A2 was from Assay BioTech (Sunnyvale, CA, USA). Secondary horseradish peroxidase-conjugated goat anti-rabbit and goat anti-mouse antibodies were from Jackson ImmunoResearch (West Grove, PA, USA). The bicinchoninic acid (BCA) protein assay kit was from Pierce (Rockford, IL, USA). Skim milk was obtained from Difco (Franklin Lakes, NJ, USA). Pentobarbital sodium (Pental) was from CTS Kiryat Malachi, Israel. Heparin sodium was from Rotexmedica (Trittau, Germany). All the other reagents were purchased from Sigma–Aldrich (Rehovot, Israel).

### Cell Culture

The Madin–Darby canine kidney (MDCK) II cells transfected with cDNA coding for MDR1 (MDCK-MDR1) and wild-type (MDCK-CT) cells were kindly provided by Dr. Alfred Schinkel (The Netherlands Cancer Institute). The human colorectal adenocarcinoma cell line HT-29 was a generous donation of Dr. Ioav Cabantchik (The Hebrew University). MDR1 was induced in HT-29 cells (HT-29-MDR1) by incubation of control HT-29 cells (HT-29-CT) with 300 ng/mL colchicine in growth medium for a minimum of 9 weeks. All cell types were grown in Dulbecco’s modified Eagle’s phenol-free low-glucose medium (DMEM) supplemented with 10% fetal bovine serum, 2 mM L-glutamine, 100 units/mL penicillin, and 100 μg/mL streptomycin at 37°C in a 5% CO_2_ incubator, and HT-29-MDR1 cells were in addition continuously incubated with colchicine.

### Animals

The experimental studies and protocols were approved by the Animal Care and Use Committee of the Hebrew University (Protocol # MD-12-13573) and the procedures followed were in accordance with institutional guidelines. Male CD1-nude-white mice (6–7 weeks old) were purchased from Harlan Laboratories (Rehovot, Israel) and housed in the specific pathogen-free facility (SPF) unit at the Ein Kerem campus of the Hebrew University. The mice, weighing 31 ± 2 g, had free access to food (a standard diet) and water and were maintained on a 12:12-h automatically timed light/dark cycle.

### Accumulation Assays

Probes chosen for these studies included two compounds previously reported to be MDR1 substrates, ICG (Portnoy et al., 2012) and rhodamine 800 (On et al., 2011), as well as several additional cyanine dyes: IR-775, IR-783, XenoLight DiR, and Genhance 750. Accumulation studies were conducted using MDCK-CT [which express endogenous, canine MDR1 (Feng et al., 2008)], MDCK-MDR1, HT-29-CT, and HT-29-MDR1 that were seeded separately in 96 well plates at 8 × 10^4^ cells/well and reached confluence, forming monolayers. The tested probe was dissolved in dimethyl sulfoxide (DMSO) and diluted in DMEM (maximal final DMSO concentration in the incubation medium 0.001%). One hundred microliter of the probe at one of nine concentrations (range 4 × 10^-7^ M–1.0 × 10^-4^ M) was added to each well of both MDCK-CT and MDCK-MDR1 plates. Following 1 h incubation with the probe, cells were washed three times with PBS and emission signal was quantified using Cytation 3 Cell Imaging Multi Mode Reader (BioTek, Winooski, VT, USA). In a separate set of experiments, control MDCK cells were incubated with IR-783 (2 × 10^-5^ M) in 24 well plates and accumulation was measured as described above at the presence or the absence of verapamil (200 μM). Plates were scanned by Typhoon FLA 9500 biomolecular imager (GE Healthcare Life Sciences, Piscataway Township, NJ, USA).

### Permeability Assays

Transport of IR-783 across cell monolayers was evaluated as described before (Portnoy et al., 2012). Briefly, MDCK-CT cells or MDCK-MDR1 cells were seeded at 2 × 10^6^ cells/well on microporous polycarbonate membrane filters. Cells were grown until transepithelial resistance reached 200 Ω or more (measured by Millicell-ERS, Millipore Corporation, Billerica, MA, USA), with daily replacement of medium. The experiments began with replacing the medium with fresh DMEM containing the probe (1.25 × 10^-5^ M). Transport of the probes was tested for both the apical to basolateral (A to B), and the basolateral to apical (B to A) directions. Aliquots of 100 μL were taken from the receiver compartments every 30 min with replacement with fresh DMEM. Probe emission intensity was measured by the Cytation 3 Reader. The studies were performed in triplicate in a humidified incubator on two different days. Papp and the ER were calculated as described before (Portnoy et al., 2012).

### *In vivo* Imaging Studies

HT-29-CT and HT-29-MDR1 xenografts were initiated by subcutaneous injection of 2.8 × 10^6^ cells into the flanks of the mice. HT-29-CT and HT-29-MDR1 cells were injected to the right and left flanks, respectively. Three weeks after grafting, mice were subjected to optical imaging. On each study day, 10 μL/gr ICG or IR-783 (8 mg/kg or 4 mg/kg, respectively, in 10 parts DDW:two parts filtered sucrose phosphate buffer 9.3%) was injected into the tail vein. Mice were repetitively scanned over a time period of 90 min, while body temperature was kept on a 37°C platform. This period was selected based on preliminary studies demonstrating that longer imaging periods do not increase the sensitivity to detect differences between control and MDR1 tumors and lead to greater mortality.

At the completion of the scans, mice were sacrificed under pentobarbital sodium anesthesia (200 mg/mL, 350 mg/kg) and cardiac blood samples (40 μL) were collected into heparinized 96-well plates. Subsequently, tumors were collected. Along the collection procedure, tissue and blood samples were protected from light and kept on ice. Immediately after that, they were scanned by the Typhoon FLA 9500 imager, to verify the results obtained *in vivo* (because the *in vivo* signal might potentially reflect emission from tissues underneath the tumor). The tissues were then frozen in liquid nitrogen and kept in -80°C until further analysis.

### Image Analysis and Data Analysis

For analysis of probe uptake kinetics *in vitro*, we calculated the area under the emission-intensity-concentration curve (AUC) using the trapezoidal rule. Larger AUC implies lower uptake or greater eﬄux of the probe. Due to non-linearity of the emission intensity with regard to substrate concentrations, E_max_ and EC_50_ values are not reported.

For analysis of the *in vivo* dye kinetics, regions of interest (ROIs) were drawn over the tumors (0.25 ± 0.1 cm^2^) using Living Image 4.3.1 (PerkinElmer, Waltham, MA, USA). A constant size ROI (0.1 cm^2^) was drawn near every tumor and used as a reference background region as recommended by the manufacturer of the *in vivo* imaging system (Caliper LifeSciences, 2012). The emission intensity was expressed in radiant efficiency units ([photons/second/steradian]/microwatt; [p/s/sr]/μW). Areas under the concentration-time curve (AUC) were calculated using Phoenix WinNonlin 6.3. The *ex vivo* emission intensity was analyzed using ImageJ 1.47V (National Institute of Health, Bethesda, MD, USA).

### Quantitative Real-Time Polymerase Chain Reaction

Analysis of mRNA levels was conducted as described before (Rubinchik-Stern et al., 2015). Briefly, total RNA was isolated using RNeasy mini-isolation kit. RNA integrity and purity were verified by ND-1000 spectrophotometer (NanoDrop Technologies, Inc). cDNA was synthesized from 2 μg of total RNA that had A260/A280 and A230/A260 ratios of 1.8–2.0 and 2.0–2.2, respectively. Reverse transcription was performed using TaqMan reverse transcription reagents. The reactions were run as follows: 25°C for 10 min, followed by 37°C for 120 min, then 85°C for 5 min. The real-time PCR assay was carried out with the use of gene specific FAM-labeled fluorescent MGB probes in StepOnePlus real-time PCR system (Applied Biosystems) on a fast mode. Samples were run in triplicate. The reaction final volume was 10 μL for each sample. The relative mRNA levels in each sample were normalized to the housekeeping gene β-actin. Changes in mRNA expression of target genes from the control cells were expressed relative to that of the vehicle control group.

### Western Blot Analysis

Several tumors were large enough to allow measurement of both the mRNA and the protein levels of MDR1. From these tumors, whole cell lysates were prepared as previously described (Rubinchik-Stern et al., 2015), by tissue homogenization in cold radioimmunoprecipitation assay (RIPA) buffer. Lysates were subjected to sodium dodecyl sulfate-polyacrylamide gel electrophoresis (SDS-PAGE). Stacking and separating gels were made of 5 and 10% acrylamide, respectively. Each lane was loaded with 20 μg protein samples of whole cell lysates. Following separation, the proteins from the gels were transferred to nitrocellulose membranes using a Mini Trans-Blot Cell (Bio-Rad Laboratories, Inc.). Membranes were blocked in Tris-buffered saline containing 0.1% Tween 20 (TBS-T) and 5% skim milk powder and probed overnight at 4°C with primary antibodies against β-actin (1:1,000), P-gp (recognizes both the human and the murine MDR1; 1:600), and OATP1A2 (SLCO1A2; 1:500). Antibodies were diluted in 5% w/v BSA in 0.1% Tris-Buffered Saline with Tween-20 and 0.02% sodium azide. The blots were then incubated for 1 h with horseradish peroxidase-conjugated secondary antibodies (goat anti-rabbit) at 1:10,000 dilutions and developed by enhanced chemiluminescence. β-Actin was used as the internal control.

### Statistical Analysis

Results are reported as mean ± SD, unless otherwise indicated. Values of MDR1 and control tumors were compared using the Wilcoxon signed rank test (InStat; GraphPad, La Jolla, CA, USA). A *p* value ≤ 0.05 was considered significant.

## Results

### *In vitro* Characterization of the NIR Probes

**FIGURE 1:**
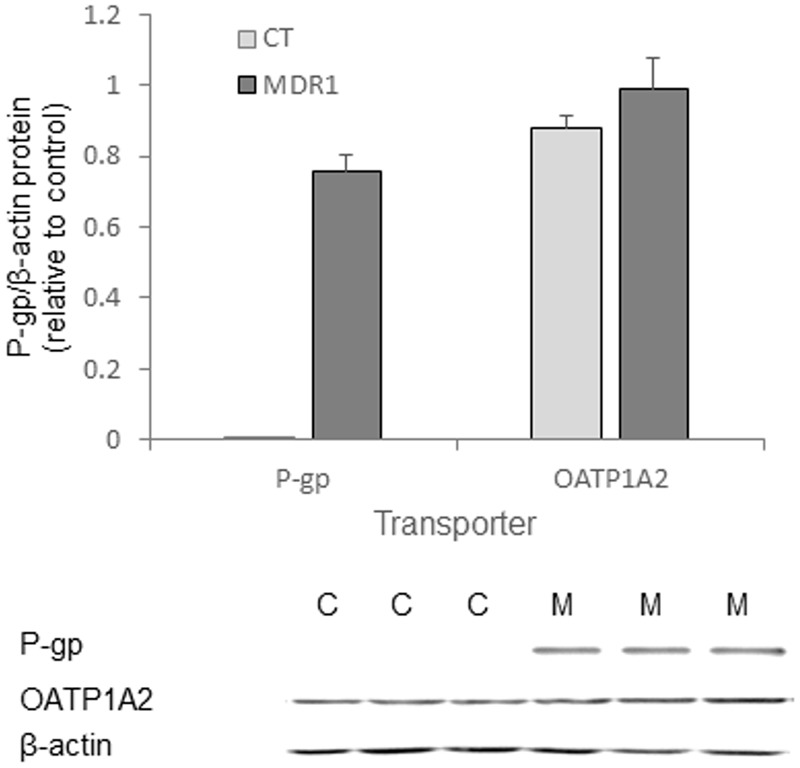
***In vitro* transporter expression in HT29-CT and HT29-MDR1 (multidrug resistance protein 1) tumors.** Shown are mean ± SD values of expression (*n* = 3) and an immunoblot of HT-29-MDR1 and HT-29-CT extracts. Mean relative transcript and protein levels in control tumors is set as 1. C, HT-29-CT cells; M, HT-29-MDR1 cells.

**FIGURE 2:**
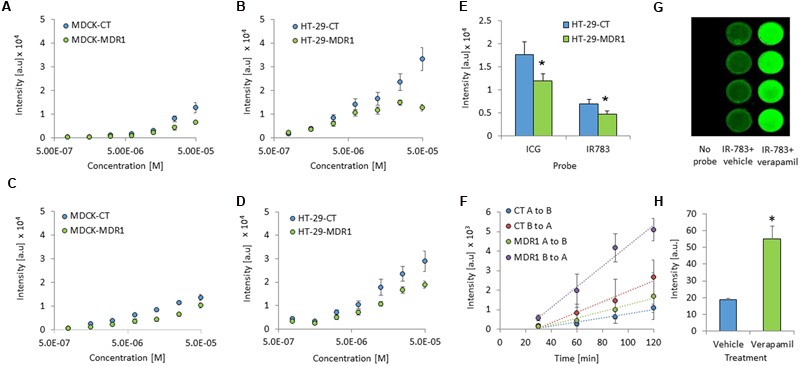
***In vitro* cellular accumulation of ICG and IR-783 in MDR1 and control cells. (A)** ICG accumulation in Madin–Darby canine kidney (MDCK)-CT and MDCK-MDR1 cells. **(B)** ICG accumulation in HT-29-CT and HT-29-MDR1 cells. **(C)** IR-783 accumulation in MDCK-CT and MDCK-MDR1 cells. **(D)** IR-783 accumulation in HT-29-CT and HT-29-MDR1. Cells were incubated for 1 h with the indicated concentrations of the tested compound (*n* = 16 per each concentration). **(E)** ICG and IR-783 accumulation in HT-29 cells at 1.25 × 10^-5^ M (mean ± SD). Fluorescence intensity is presented as arbitrary units (a.u). **(F)** Transfer of IR-783 across MDCK-CT and MDCK-MDR1 cell monolayers. **(G)** An image of control MDCK cells incubated with IR-783 (2 × 10^-5^ M) at the presence or the absence of verapamil (200 μM). The experiment was repeated twice, on two different days. **(H)** Emission intensity of control MDCK cells following incubation with IR-783 as described for (G; *n* = 8). ^∗^*p* < 0.01.

MDR1 overexpression in HT-29-MDR1 cells was confirmed by western blot analysis. This analysis further demonstrated that the expression of a representative uptake transporter, OATP1A2 is not significantly altered in these cells (**Figure 1**). Among the tested compounds, XenoLight and GenHance accumulated in MDCK-CT cells only scarcely, whereas rhodamine 800 and IR-775 demonstrated a quenching phenomenon at high concentrations (Mann et al., 2015). ICG and IR-783 accumulation increased with the dye concentration in all cell types. Both ICG and IR-783 appeared to accumulate to a lesser extent in MDR1-overexpressing MDCK and HT-29 cells than in their respective controls [**Table 1**; **Figures 2A–D**; see also (Portnoy et al., 2012)]. At 1.25 × 10^-5^ M, the emission intensity of both ICG and IR-783 in HT-29-CT cells was 1.5-fold (*P* < 0.01) greater compared to HT-29-MDR1 cells (**Figure 2E**). The ERs of IR-783, calculated from the permeability assays, were 1.2 and 2.0 in MDCK-CT and MDCK-MDR1 cells, respectively, resulting in a net ER of 1.7 (**Figure 2F**). At the presence of verapamil, the emission intensity of control MDCK cells incubated with IR-783 was 2.9-fold higher than in cells incubated with the vehicle (**Figures 2G,H**). The ER of ICG and the effect of verapamil on its accumulation have been published before (Portnoy et al., 2012). Because ICG and IR-783 have shown the best imaging properties among the probes studied *in vitro*, they were selected for farther evaluation.

**Table 1:** AUC of ICG and IR-783 in control and multidrug resistance protein 1 (MDR1)-overxpressing Madin–Darby canine kidney (MDCK) and HT-29 cells.

Compound	AUC			
	MDCK cells		HT-29 cells	
	MDCK-CT	MDCK-MDR1	HT-29-CT	HT-29-MDR1
ICG	1.2	0.6	2.9	1.3
IR-783	1.3	0.9	2.6	1.8

### *In vivo* Studies

**FIGURE 3:**
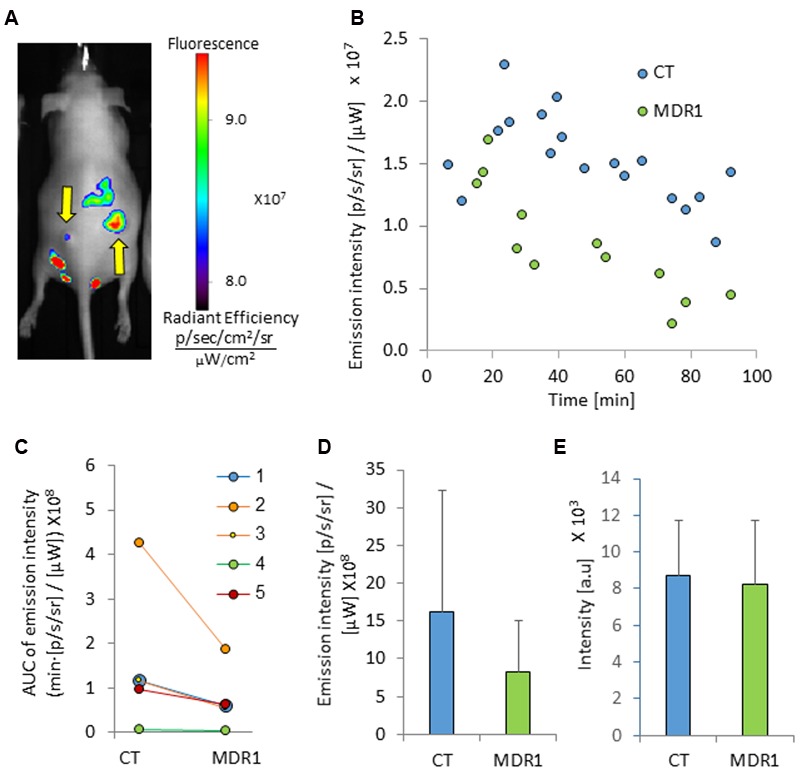
**Tumor emission intensity in mice treated with ICG. (A)** An image of a mouse bearing control (right) and MDR1 (left) tumors treated with intravenous 8 mg/kg ICG (indicated by arrows). The time from ICG injection to image acquisition was 74 min. **(B)** A representative emission intensity-time curve in HT-29-CT and HT-29-MDR1 tumors of the same mouse. **(C)** Individual AUC values of HT29-CT and HT29-MDR1 tumor emission intensity in ICG-treated mice. The AUC was calculated over the first 90 min after ICG injection. **(D)** AUC values of *in vivo* control and MDR1 tumor emission intensity (*n* = 5). **(E)** ICG emission from of *ex vivo* control and MDR1 tumors. Results are mean ± SD.

*In vivo*, MDR1 tumors did not significantly differ in size from control tumors, although variability in size across animals was observed for both tumor types (**Table 2**). Optical imaging of mice treated with ICG demonstrated tumor-specific signal accumulation (**Figure 3**). Elevated emission intensity in tumors located on the right flanks of the mice indicated higher ICG concentrations in HT-29-CT tumors, compared to their opposing HT-29-MDR1 tumors (**Figures 3A,B**). The AUC of emission intensity calculated individually for each mouse was greater in CT compared to MDR1 tumors, with individual CT/MDR1 AUC ratios of 2.2, 2.0, 1.8, 1.5, and 1.3 (mean 1.7; **Figure 3C**). The difference in emission intensity between the two tumor types were not significant *in vivo* or *ex vivo* (*p* > 0.05; **Figures 3D,E**).

**Table 2:** Tumor sizes on the imaging study date.

	Tumor area (cm^2^)			
	ICG-treated mice		IR-783-treated mice	
Mouse number	Control tumors	MDR1 tumors	Control tumors	MDR1 tumors
1	0.80	0.55	0.57	0.24
2	0.29	0.32	0.65	0.08
3	0.57	0.31	0.35	0.22
4	0.13	0.32	0.25	0.16
5	0.23	0.25	0.19	0.78
Mean	0.40	0.35	0.40	0.30
*SD*	0.28	0.16	0.20	0.28

**FIGURE 4:**
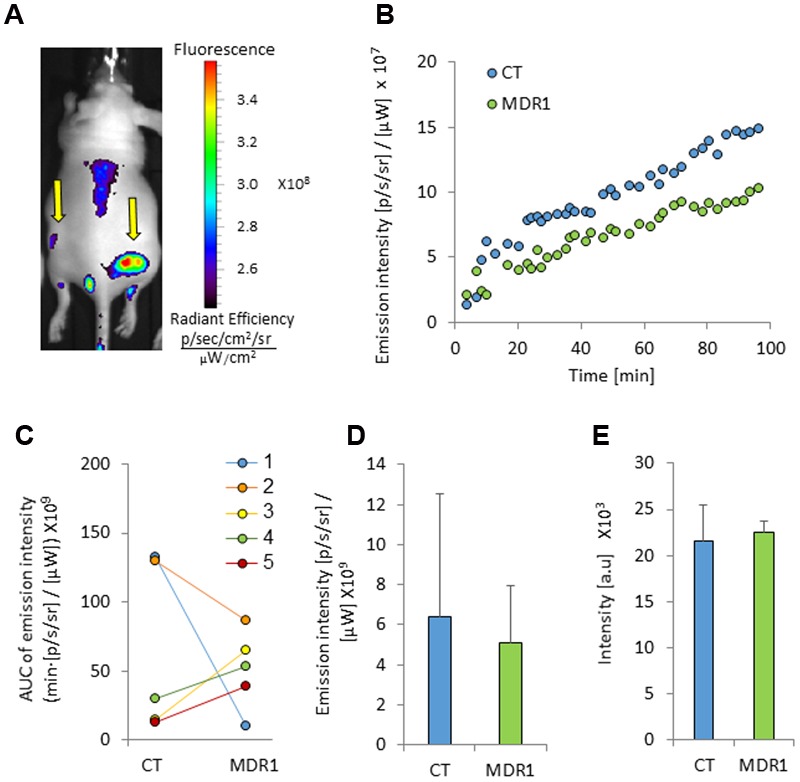
**Tumor emission intensity in mice treated with IR-783. (A)** An image of a mouse bearing control and MDR1 tumors treated with intravenous 4 mg/kg IR-783 (indicated by arrows). The time from ICG injection to image acquisition was 76 min. **(B)** An emission intensity-time curve in HT-29-CT and HT-29-MDR1 tumors in the same mouse. **(C)** Area under the emission intensity-time curve (AUC) values of HT29-CT and HT29-MDR1 tumors in IR-783-treated mice. The AUC was calculated over the first 90 min after ICG injection. **(D)** AUC values of IR-783 *in vivo* emission intensity in control and MDR1 tumors (*n* = 4). **(E)** IR-783 emission from *ex vivo* control and MDR1 tumors. Shown are mean ± SD.

IR-783 accumulated mostly in the largest control tumors (0.57 cm^2^, 0.65 cm^2^). In mice which bared these tumors the probe accumulation appeared to be greater in control versus MDR1 tumors (**Figure 4**). However, in mice baring smaller control tumors (0.08 cm^2^, 0.22 cm^2^, 0.16 cm^2^), IR-783 emission intensity was greater in MDR1 than in control tumors (**Figure 4C**). Hence, the mean IR-783 did not differ between the tumor types (**Figures 4D,E**). No relationship was observed between MDR1 tumor size and emission intensity (data not shown).

### Transporter Expression

**FIGURE 5:**
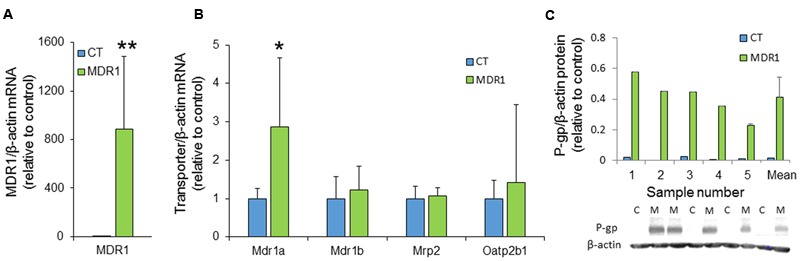
**MDR1 expression in HT29-CT and HT29-MDR1 tumors. (A)** MDR1 mRNA levels in tumor xenografts from CT and MDR1 HT-29 cells. **(B)** mRNA levels of additional transporters that may be involved in ICG and IR-783 tumor accumulation. Note the differences in the Y-scale between **(A)** and **(B)**. Wilcoxon paired test, ^∗^*p* < 0.05, ^∗∗^*p* < 0.001. **(C)** MDR1 protein expression in tumors resected from ICG-treated (*n* = 4) and IR-783-treated (*n* = 1) mice. The lower panel demonstrates an immunoblot of HT-29-MDR1 and HT-29-CT xenograft extract. Mean relative transcript and protein levels in control tumors was set as 1. Also shown are the mean ±*SD* values of expression. C, HT-29-CT tumors; M, HT-29-MDR1 tumors.

RT-PCR analysis of xenograft extracts demonstrated significantly elevated MDR1 mRNA levels in HT-29-MDR1 tumors, indicating that these cells maintained MDR1 overexpression *in vivo* (**Figure 5A**). Interestingly, the expression of the murine Mdr1a that might contribute to ICG and IR-783 accumulation differed between control and the MDR1 xenografts (**Figure 5B**). However, the magnitude of difference was well below that observed for MDR1. Western blotting of proteins from tumors that were large enough to provide material for both mRNA and protein expression analyses confirmed MDR1 overexpression at the protein level (∼30-fold greater than in control tumors; **Figure 5C**). No correlation was found between MDR1 expression (at either the mRNA or the protein level) and ICG emission intensity (data not shown).

## Discussion

Molecular imaging technologies are being increasingly used for the non-invasive assessment of membrane transporter expression and function in animal models and in humans. Among these, PET and SPECT are being applied in the clinic. However, both PET and SPECT involve health risk and are technically demanding (Mann et al., 2015, 2016). In this study we evaluated for the first time the feasibility of utilizing an alternative method, NIR, for assessing MDR1-mediated eﬄux transport in tumors *in vivo*. Our results demonstrate the advantages and challenges of this methodology.

Many NIR probes are characterized by poor photostability, high plasma protein binding, and aggregation and quenching at high concentrations (Mann et al., 2015, 2016). Indeed, some of the compounds that we initially evaluated through accumulation assays were excluded from further analysis because of our concern that potential accumulation in tumors may lead to quenching of their fluorescence. The remaining compounds, ICG (Portnoy et al., 2012) and IR-783, had favorable accumulation profiles and their accumulation was enhanced by the MDR1 inhibitor verapamil. Hence, these probes were selected for further analysis. However, the *in vivo* studies demonstrated low sensitivity of the probes in detecting MDR1 activity in tumor xenografts. ICG’s MDR1:control tumor emission intensity ratio was 1.7 only, as compared to a 6.8-fold difference in the probe’s ER *in vitro* (Portnoy et al., 2012), and the results obtained with IR-783 were highly variable. The scaled-down sensitivity could represent MDR1 saturation *in vivo*. In addition, ICG is a marker of blood flow, which could potentially differ between control and MDR1 tumors. This could have led to attenuation (or enhancement) of the changes in MDR1 activity as measured by emission intensity (Mann et al., 2016). Furthermore, variation in blood flow between larger and smaller tumors could have contributed to the between-subject variability in IR-783 emission. Yet, it should be kept in mind that even established markers of MDR1 activity have limited sensitivity and high between-subject variability when they are used for detecting MDR1-mediated MDR in solid tumors. For instance, breast cancer tumors expressing high amounts of MDR1 displayed only a 2.7-fold higher [^99m^Tc]-sestamibi eﬄux rate compared to tumors expressing little or no MDR1 (Del Vecchio et al., 2003). When [^11^C]-verapamil was administered to soft tissue sarcoma patients along with markers of cellular proliferation and hypoxic volume, [^11^C]-radioactivity in tumors varied between patients and with respect to the uptake parameters of other probes in individual patients (Eary et al., 2011). In patients with advanced lung cancer, [^11^C]-docetaxel radioactivity was moderate and highly variable between and within tumors (van der Veldt et al., 2013).

Several limitations should be noted. Particularly, the small animal numbers could have limited our ability to identify differences in probe uptake between MDR1 and control tumors. However, with the exception of one IR-783-treated animal, the magnitude of difference in emission intensity between MDR1 and control tumors *in vivo* was low. We therefore concluded that the selected probes were not optimal for *in vivo* identification of MDR1-mediated drug resistance and did not pursue using these compounds *in vivo* (e.g., with larger animal numbers and MDR1 inhibitors). This decision was made given that in clinic, tumors are expected to present an entire spectrum of MDR1 expression rather than a binomial distribution and detection of MDR1 activity would be further challenging. We used HT-29 cell xenografts as the *in vivo* tumor model. Such subcutaneous xenografts have limitations, such as the lack of appropriate tumor microenvironment and selection for clones that are no longer representative of the original tumor. However, these models have identified clinically efficacious agents (Gura, 1997; Morton and Houghton, 2007) and the tumor subcutaneous localization is advantageous for optical imaging. We have also validated MDR1 expression in these tumors at both the mRNA and the protein levels, although MDR1 functionality was not proven and the level of several additional transporters varied between control and MDR1 tumors (albeit to a much lesser extent than that of MDR1). Although we inoculated similar numbers of HT-29-CT and HT-29-MDR1 cells into each mouse, the growth rate and subsequent blood flow could have differed between MDR1 and control tumors, as described above. Finally, ICG and IR-783 are substrates of transporters other than MDR1. In particular, OATPs mediate the uptake of both compounds into tumor cells (Yang et al., 2010; de Graaf et al., 2011). However, the differences between control and MDR1 tumors in the expression of these transporters were negligible, as compared to that in MDR1 expression.

Despite the above mentioned limitations, this study has several important strengths. First, each mouse was used as its own control for evaluating the impact of MDR1 on the probe emission from the tumors. Hence, normalization to the injected dose or plasma concentrations was unnecessary. Second, we verified tumor MDR1 expression (or paucity thereof) in the inoculated tumors at both the mRNA and the protein level. Finally, we provided a proof of concept for the existence of methodology that will allow future visualization of MDR1 activity in tumor cells *in vivo*, using other NIR probes.

## Conclusion

This study evaluated for the first time the feasibility of detecting MDR1-mediated MDR by the use of NIR imaging. *In vitro*, the evaluated probes were poorer MDR1 substrates, or did not behave as substrates, as compared to fluorescent compounds which are detectable at lower wavelengths. Initial results with probes that were assessed *in vivo* demonstrate low sensitivity to identify tumor MDR1 activity. However, screening of additional compounds or targeted synthesis may yield better MDR1 substrate probes that can provide a tool for non-radioactive identification of MDR1-mediated drug resistance in solid tumors. With further optimization, such methodologies could be useful markers for drug selection and prognosis when the tumors or their metastases are not deep (e.g., breast tumors and lymph node metastases) or for tumors that can be monitored by endoscopy. In the meantime, newly synthesized NIR probes might be screened for their interaction with P-gp and other eﬄux transporters prior to their use *in vivo*, especially if the probes are intended for studying tumor biology.

## Author Contributions

IS and EP conducted the *in vivo* studies and some of the *in vitro* analyses; IS wrote the manuscript; MA, MS, and GI conducted the *in vitro* studies; SG developed the models used for analyses of the *in vitro* data; SE supervised the work and manuscript preparation. All authors revised the manuscript.

## Conflict of Interest Statement

The authors declare that the research was conducted in the absence of any commercial or financial relationships that could be construed as a potential conflict of interest.

## Abbreviations

DMEM: Dulbecco’s modified Eagle’s medium
ER: eﬄux ratio
ICG: indocyanine green
NIR: near infrared
OATP: organic anion transporting polypeptide
Papp: permeability coefficient
PET: positron emission tomography
SPECT: single-photon emission computed tomography
